# Evaluating User Engagement and Satisfaction With Digital Mental Health Interventions: Randomized Controlled Trial of a Text Messaging Program and e-Mental Health Resources

**DOI:** 10.2196/76587

**Published:** 2026-01-21

**Authors:** Gloria Obuobi-Donkor, Reham Shalaby, Belinda Agyapong, Samuel Obeng Nkrumah, Medard Kofi Adu, Ejemai Eboreime, Lori Wozney, Vincent Israel Opoku Agyapong

**Affiliations:** 1 Department of Psychiatry, Faculty of Medicine, Dalhousie University, 5909 Veterans Memorial Lane, 8th Floor. Abbie J. Lane Memorial Building QEII Health Sciences Centre, Halifax, NS, Canada, 1 7802157771; 2 Department of Psychiatry, Faculty of Medicine & Dentistry, University of Alberta, Edmonton, AB, Canada; 3 Mental Health and Addictions Program, IWK Health, Halifax, NS, Canada

**Keywords:** text messaging, online resources, mental health, Nova Scotia, user satisfaction

## Abstract

**Background:**

Digital mental health tools, such as SMS text messaging and online resources, are increasingly used to support well-being. However, user satisfaction across these formats remains insufficiently explored.

**Objective:**

The study assessed participants’ engagement, perceived impact, and overall satisfaction with the Text4Support program and the e-mental health resources.

**Methods:**

This randomized controlled study was conducted in Nova Scotia, Canada. Participants were assigned to either the Text4Support group, which received daily supportive text messages, or the control group, which received a single text message with a link to the Nova Scotia Mental Health and Addiction Program e-mental health resources. Responses to various aspects of the interventions were evaluated using a 5-point Likert scale, while overall satisfaction was measured on a scale from 0 to 10. The chi-square test and Fisher exact test were employed for data analysis.

**Results:**

A total of 69 participants in the control group and 130 in the Text4Support group completed the satisfaction survey. The overall mean (SD) satisfaction score in the control group was 5.1 (2.3), and the overall mean (SD) satisfaction score for the Text4Support group was 7.1 (2.2). Compared to the control group, participants in the Text4Support group reported greater engagement and positive program impact. While 53.8% (70/130) of Text4Support recipients always read the messages, only 39.1% (27/69) of the control group rarely accessed the eHealth resources. When compared to the control group, participants allocated to the Text4Support group were reported to sometimes take positive action upon reading the messages (42.3% vs 33.3%). A significantly higher proportion of Text4Support users strongly agreed or agreed that the messages were supportive (81.4% vs 41.5%), positive (88.4% vs 49.2%), and helpful in coping with stress (44.2% vs 11.9%), loneliness (40.3% vs 13.4%), and improving mental well-being (51.2% vs 17.9%). In contrast, the majority of responses from the control group were largely neutral.

**Conclusions:**

Results showed that Text4Support group participants were significantly more satisfied with the program than those receiving standard eHealth resources. This highlights that daily supportive SMS text messaging is an effective, low-cost adjunct to care delivery and mental health improvement. These findings suggest that aggregate, brief, and low-cost text-based interventions have great potential for increasing health access and engagement, particularly among traditionally disadvantaged populations with limited access to traditional services.

## Introduction

Psychiatric care has increasingly incorporated digital mental health interventions, which offer accessible and scalable support beyond traditional therapy and medication. Patient feedback through user satisfaction measures is vital in assessing the effectiveness of digital mental health interventions [1]. Patient experiences are gaining recognition as a key factor in enhancing health care quality alongside clinical effectiveness and safety [2,3]. These patient care experiences are included in public reporting and health performance programs [3]. Supportive SMS text messaging programs and eHealth services have become popular and convenient tools for managing mental health [4,5]. The structure and approaches of these interventions may vary in terms of user engagement and perceived effectiveness, but all aim to enhance well-being [6]. Nevertheless, satisfaction levels often vary, highlighting an area that requires more attention to optimize the effectiveness of such interventions for individuals with mental illness.

Supportive SMS text messaging programs like Text4Hope, Text4Mood, Text4PTSI, and Wellbeing offer automated text messages that provide preprogrammed positive reinforcement to users [7-9]. These coping message text programs require minimal input from users while guiding users through daily coping skills and emotional support [7-11]. Literature has shown that these programs increase feelings of connectedness and reduce mental health conditions [10]. Two randomized controlled trials (RCTs) conducted in Canada revealed that supportive text messages reduced depression and alcohol intake of subscribers [12,13].

Various studies have shown that eHealth resources such as online self-help services, psychoeducational tools, and virtual peer support groups provide an accessible way to seek help, as well as an independent role in receiving care [14,15]. These entail being able to partake in the content through specific timestamps, which ensures self-governance [16]. Nevertheless, there is little standardization regarding how users engage with eHealth interventions; it remains their prerogative to seek and employ the information furnished, and this influences overall satisfaction and perceived efficacy.

. This study aims to compare supportive SMS text messaging interventions with eHealth resources to better understand users’ perceptions and feedback on the impact, engagement levels, and overall satisfaction. The study will evaluate engagement and user satisfaction with Text4Support in comparison to eHealth resources and assess the perceived relevance and impact of supportive SMS text messaging relative to these eHealth tools.

Given the importance of the perceived adequacy of digitally delivered tools as a core component of modern psychiatric intervention delivery systems to their success, it is essential to understand how users evaluate the adequacy of digitally delivered interventions. This study aims to compare supportive SMS text messaging interventions with eHealth resources to better understand users’ perceptions and feedback on the impact, engagement levels, and overall satisfaction. The study will evaluate engagement and user satisfaction with Text4Support in comparison to eHealth resources and assess the perceived relevance and impact of supportive SMS text messaging relative to these eHealth tools.

The intervention, Text4Support, provides daily cognitive behavioral therapy (CBT)-informed supportive text messages for 6 months, tailored to users’ primary mental health concerns and delivered as an adjunct to usual care. In contrast, the control group receives the usual care plus a single text message directing participants to freely available e-mental health tools offered through the Nova Scotia Mental Health and Addictions Program website.

## Methods

### Study Design

This RCT adopted a multicenter, two-arm design with a rater-blinded methodology. Participants were recruited and randomized into the Text4Support group and the control group.

### Ethical Considerations

The study was approved by the Nova Scotia Health Research Ethics Board (REB File #1028174) and registered with ClinicalTrials.gov (NCT05411302). Each study participant provided informed consent, and the study was conducted according to the Declaration of Helsinki [17] and Good Clinical Practice (Canadian Guidelines) [18]. Participation was voluntary, with the option to withdraw at any time without impact on care. Participants’ privacy and confidentiality were rigorously protected. All data were deidentified at the point of collection, stored, and secured. No compensation or incentives were provided to participants.

### Data Collection/Intervention

The published study protocol detailed the data collection procedure [19]. In summary, data were collected via a self-administered survey powered by the Research Electronic Data Capture (REDCap) software program [20]. Participants’ age, gender, income, education, housing status, relationship status, and clinical information were obtained. Data were collected between October 11, 2022 and July 31, 2024. After thoroughly informing participants about the study, we used an electronic consent form to obtain their consent.

Participants randomized to the control group received one text message with the Nova Scotia Mental Health and Addictions Program website link embedded in the message. This resource was freely accessible, and this service was provided as an add-on to participants’ usual care. The website offers free, evidence-based e-mental health resources designed to address various psychiatric conditions with programs such as the early psychosis program, the eating disorder program, and recovery support [21]. Participants randomized to the Text4Support group received free daily, unilateral, CBT-based text messages tailored to their specific diagnosis as an add-on program to their usual care for 6 months. The supportive text messages were developed using evidence-based principles of CBT. Each message, limited to 160 characters, was designed to provide brief, accessible prompts that encourage adaptive coping, cognitive reframing, and emotional regulation. The content was collaboratively created by CBT therapists, mental health professionals, and individuals with lived experience to ensure both clinical relevance and user-centered design. The underlying mechanism is grounded in the concept that regular exposure to concise, positively framed messages can reinforce helpful thinking patterns, enhance self-efficacy, and foster emotional resilience over time. Through consistent daily reinforcement, these messages aim to augment usual care by promoting psychological well-being and perceived connectedness. Participants completed the baseline surveys on enrollment via the online link. A text message with follow-up surveys, which includes the satisfaction survey, was sent to all participants at 6 weeks, 3 months, and 6 months.

### Outcome Measures

The primary outcome measure was the participants’ overall satisfaction with the Text4Support program or the Nova Scotia Health e-mental health online resources. Overall satisfaction was rated on an 11-point Likert scale, where zero indicated “very dissatisfied,” 5 indicated “neutral,” and 10 showed “very satisfied.”

Secondary outcomes included the perceived impact and feedback on the Text4Support program and the Nova Scotia Health e-mental health online resources among the Text4Support and control groups. Study participants provided feedback on their satisfaction by responding to questions that assessed their engagement and use of each intervention and its perceived impact.

Engagement and use of each intervention were measured on a 5-point Likert scale (always, mostly, sometimes, rarely, and never). The perceived impact of each intervention on how participants coped with stress and loneliness, improved quality of life, overall physical and mental well-being, and the intervention's relevance, encouragement, and supportiveness were measured on a 5-point Likert scale, ranging from “strongly agree” to “strongly disagree” and including “neutral.” For analysis purposes, responses were collapsed into 3 categories: strongly agree/agree, neutral, and strongly disagree/disagree.

The reliability and validity of this scale for testing satisfaction have not been examined; however, the survey was adopted in various studies to assess user satisfaction [8,10,22].

### Data Analyses

Data were analyzed using SPSS for Windows (version 28; IBM Corporation) [23]. Participants’ satisfaction data were presented as a continuous variable. We measured participants’ overall satisfaction by rating the program from 0 to 10 (0=very dissatisfied, 5=neutral, and 10=very satisfied). The results were presented as means and SDs. Participants’ engagement and use of each intervention (Text4Support/online resources) and the perceived impact of each intervention were summarized and presented as categorical variables and reported as frequency and percentages among the control group and Text4Support group. A chi-square and Fisher exact test were run to examine any differences in reporting user satisfaction, engagement, or perception of the Text4Support or online resources among the 2 groups, and a 2-tailed criterion (*α*<.05) was used to determine statistical differences. To handle missing data, we adopted imputation techniques, focusing on the last observation carried forward method.

## Results

**Figure 1.**
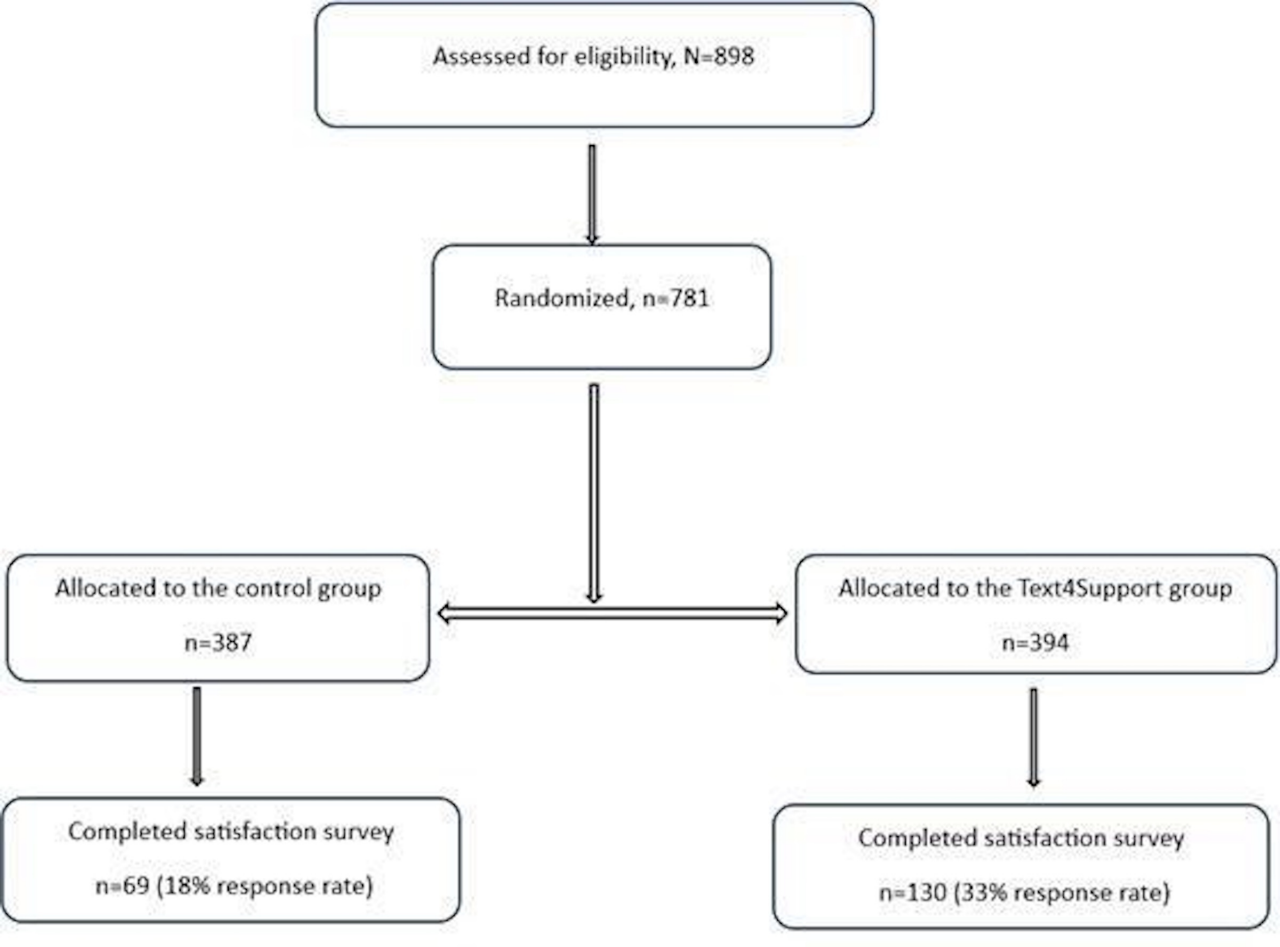
Participant flowchart.

Figure 1 illustrates the flowchart of the participants. Eight hundred and ninety-eight patients were assessed for eligibility to enter the trial, of whom 781 eligible patients were randomized: 69 in the control group and 130 in the Text4Support group completed the satisfaction survey. The response rate in the control group was 18% (69/387), whereas the Text4Support group achieved a higher response rate of 33% (130/394).

Table 1 shows the demographic characteristics of participants who completed the satisfaction survey. In total, across the 2 groups, the majority were between 26 and 60 years old (72.9%, 145/199), female (66.8%, 133/199), Caucasian (84.8%, 168/198), employed (52.3%, 103/197), and partnered/married (45.2%, 90/199) and had obtained a postsecondary education at a college or university (57.1%, 113/198). About 49% (97/199) of the study participants live in rented accommodation, 28.5% (55/193) have an income range of less than $29,590 (1 CAD≈US $0.77 at the time of the study), and 23.1% (27/117) have a primary diagnosis of depression. The chi-square analysis revealed no statistically significant differences in the demographic characteristics of participants in the Text4Support group and the control group, suggesting no differences in their demographic characteristics.

**Table 1.** Demographic characteristics of participants who completed the satisfaction survey.

Variable	Control group, n (%)	Text4Support group, n (%)	Total, n (%)	χ^2^/Fisher exact *(df)*	*P* value
Age (y)					
≤25	13 (18.8)	21 (16.2)	34 (17.1)	3.1 (3)	.40
26‐40	29 (42.0)	44 (33.8)	73 (36.7)		
41‐60	23 (33.3)	49 (37.7)	72 (36.2)		
>60	4 (5.8)	16 (12.3)	20 (10.1)		
Sex at birth					
Male	21 (30.4)	45 (34.6)	66 (33.2)	0.4 (1)	.60
Female	48 (69.6)	85 (65.4)	133 (66.8)		
Ethnicity					
Caucasian	54 (78.3)	114 (88.4)	168 (84.8)	6.0^a^	.10
Indigenous	8 (11.6)	4 (3.1)	12 (6.1)		
Black/Hispanic	4 (5.8)	7 (5.4)	11 (5.6)		
Asian	3 (4.3)	4 (3.1)	7 (3.5)		
Employment status					
Student	7 (10.1)	11 (8.6)	18 (9.1)	2.3 (4)	.70
Employed	35 (50.7)	68 (53.1)	103 (52.3)		
Unemployed	16 (23.2)	22 (17.2)	38 (19.3)		
Retired	7 (10.1)	13 (10.2)	20 (10.2)		
Other	4 (5.8)	14 (10.9)	18 (9.1)		
Educational status					
Elementary school	2 (2.9)	2 (1.6)	4 (2.0)	1.9^a^	.80
High school	20 (29.0)	39 (30.2)	59 (29.8)		
Postsecondary (college/university)	41 (59.4)	72 (55.8)	113 (57.1)		
Postsecondary (trade school)	6 (8.7)	13(10.1)	19 (9.6)		
Other	0 (0.0)	3 (2.3)	3 (1.5)		
Relationship status					
Single	31 (44.9)	54 (42.5)	85 (42.7)	2.7^a^	.60
Partnered/married	32 (46.4)	58 (44.6)	90 (45.2)		
Separated/divorced	4 (5.8)	13 (10.0)	17 (8.5)		
Widowed	2 (2.9)	2 (1.5)	4 (2.0)		
Other	0 (0.0)	3 (2.3)	3 (1.5)		
Housing status					
Own home	19 (27.5)	43 (33.1)	62 (31.2)	1.7 (2)	.40
Rented accommodation	38 (55.1)	59 (45.4)	97 (48.7)		
Live with family/friends	12 (17.4)	28 (21.5)	40 (20.1)		
Income range (stats Canada) (1 CAD≈US $0.77 at the time of the study)					
Less than $29,590	21 (32.3)	34 (26.6)	55 (28.5)	6.1 (5)	.30
$29,591-$59,180	22 (33.8)	28 (21.9)	50 (25.9)		
$59,181-$93,000	10 (15.4)	35 (27.3)	45 (23.3)		
$93,001-$150,000	5 (7.7)	12 (9.4)	17 (8.8)		
More than $150,000	4 (6.2)	10 (7.8)	14 (7.3)		
No income	3 (4.6)	9 (7.0)	12 (6.2)		
Primary diagnosis					
Depression	7 (15.2)	20 (28.2)	27 (23.1)	8.0^a^	.30
Anxiety	6 (13.0)	17 (23.9)	23 (19.7)		
Bipolar disorder	9 (19.6)	7 (9.9)	16 (13.7)		
Substance use disorder	2 (4.3)	3 (4.2)	5 (4.3)		
Alcohol use disorder	2 (4.3)	3 (4.2)	5 (4.3)		
Trauma-related disorders	9 (19.6)	7 (9.9)	16 (13.7)		
Psychosis	3 (6.5)	4 (5.6)	7 (6.0)		
Other	8 (17.4)	10 (14.1)	18 (15.4)		

a Fisher exact values.

The overall mean (SD) satisfaction score in the control group was 5.1 (2.3), and the overall mean (SD) satisfaction score for the Text4Support group was 7.1 (2.2).

Table 2 outlines participants’ engagement with the Text4Support program and online mental health resources and the extent to which participants strongly agreed, agreed, were neutral, strongly disagreed, or disagreed with statements regarding these 2 resources. The majority of participants in the control group rarely read the eHealth resources (27/69, 39.1%), and 53.8% (70/130) in the Text4Support group always read the messages; 33.3% (23/69) in the control group sometimes takes a positive action after reading the online resources, and it was 42.3% (55/130) in the Text4Support group.

**Table 2.** Participants’ feedback and perception of the supportive text messages and eHealth resources.

Perception and feedback	Control group, n (%)	Text4Support group, n (%)	Total, n (%)	χ^2^/Fisher exact *(df)*	*P* value
Reading the messages/eHealth resources					
Always	6 (8.7)	70 (53.8)	76 (38.2)	79.8 (4)	<.001
Mostly	5 (7.2)	33 (25.4)	38 (19.1)		
Sometimes	19 (27.5)	16 (12.3)	35 (17.6)		
Rarely	27 (39.1)	6 (4.6)	33 (16.6)		
Never	12 (17.4)	5 (3.8)	17 (8.5)		
Read the messages/eHealth resources and take positive action after					
Always	3 (4.3)	13 (10.0)	16 (8.0)	15.5^a^	.003
Mostly	7 (10.1)	26 (20.0)	33 (16.6)		
Sometimes	23 (33.3)	55 (42.3)	78 (39.2)		
Rarely	21 (30.4)	28 21.5)	49 (24.6)		
Never	15 (21.7)	8 (6.2)	23 (11.6)		
The messages/eHealth resources were on the topic					
Strongly agree/agree	30 (46.2)	105 (81.4)	135 (69.6)	26.4^a^	<.001
Neutral	32 (49.2)	19 (14.7)	51 (26.3)		
Strongly disagree/disagree	3 (4.6)	5 (3.9)	8 (4.1)		
The messages/eHealth resources were on point					
Strongly agree/agree	29 (44.6)	104 (80.6)	133 (68.6)	25.7^a^	<.001
Neutral	32 (49.2)	21 (16.3)	53 (27.3)		
Strongly disagree/disagree	4 (6.2)	4 (3.1)	8 (4.1)		
The messages/eHealth resources were supportive					
Strongly agree/agree	27 (41.5)	105 (81.4)	132 (68.0)	31.7 (2)	<.001
Neutral	30 (46.2)	18 (14.0)	48 (24.7)		
Strongly disagree/disagree	8 (12.3)	6 (4.7)	14 (7.2)		
The messages/eHealth resources were positive					
Strongly agree/agree	32 (49.2)	114 (88.4)	146 (75.3)	38.0^a^	<.001
Neutral	30 (46.2)	10 (7.8)	40 (20.6)		
Strongly disagree/disagree	3 (4.6)	5 (3.9)	8 (4.1)		
The messages/eHealth resources improved my quality of life					
Strongly agree/agree	8 (11.9)	47 (36.4)	55 (28.1)	13.7 (2)	.001
Neutral	43 (64.2)	55 (42.6)	98 (50.0)		
Strongly disagree/disagree	16 (23.9)	27 (20.9)	43 (21.9)		
The messages/eHealth resources improved my overall mental well-being					
Strongly agree/agree	12 (17.9)	66 (51.2)	78 (39.8)	22.6 (2)	<.001
Neutral	40 (59.7)	38 (29.5)	78 (39.8)		
Strongly disagree/disagree	15 (22.4)	25 (19.4)	40 (20.4)		
The messages/eHealth resources improved my overall physical well-being					
Strongly agree/agree	7 (10.4)	28 (21.7)	35 (17.9)	4.5 (2)	>.99
Neutral	40 (59.7)	61 (47.3)	101 (51.5)		
Strongly disagree/disagree	20 (29.9)	40 (31.0)	60 (30.6)		
The messages/eHealth resources helped me cope with loneliness					
Strongly agree/agree	9 (13.4)	52 (40.3)	61 (31.1)	16.3 (2)	<.001
Neutral	38 (56.7)	43 (33.3)	81 (41.3)		
Strongly disagree/disagree	20 (29.9)	34 (26.4)	54 (27.6)		
The messages/eHealth resources helped me cope with stress					
Strongly agree/agree	8 (11.9)	57 (44.2)	65 (33.2)	20.8 (2)	<.001
Neutral	40 (59.7)	47 (36.4)	87 (44.4)		
Strongly disagree/disagree	19 (28.4)	25 (19.4)	44 (22.4)		
The messages/eHealth resources were not relevant to my concern					
Strongly agree/agree	13 (20.0)	12 (9.3)	25 (12.9)	24.4 (2)	<.001
Neutral	33 (50.8)	31 (24.0)	64 (33.0)		
Strongly disagree/disagree	19 (29.2)	86 (66.7)	105 (54.1)		
The messages/eHealth resources were not helpful					
Strongly agree/agree	8 (12.3)	9 (7.0)	17 (8.8)	31.4 (2)	<.001
Neutral	34 (52.3)	22 (17.1)	56 (28.6)		
Strongly disagree/disagree	23 (35.4)	98 (76.0)	121 (62.4)		
The messages/eHealth resources were not encouraging					
Strongly agree/agree	11 (16.9)	11 (8.5)	22 (11.3)	40.9 (2)	<.001
Neutral	33 (50.8)	17 (13.2)	50 (25.8)		
Strongly disagree/disagree	21 (32.3)	101 (78.3)	122 (62.9)		
The messages/eHealth resources were negative					
Strongly agree/agree	4 (6.2)	2 (1.6)	6 (3.1)	47.4^a^	<.001
Neutral	36 (55.4)	15 (11.6)	51 (26.3)		
Strongly disagree/disagree	25 (38.5)	112 (86.8)	137 (70.6)		

a Fisher exact values.

Most study participants were neutral when asked if the eHealth resources were on the topic (32/65, 49.2%), on point (32/65, 49.2%), supportive (30/65, 46.2%), helpful in coping with loneliness (38/67, 56.7%), and helpful in coping with stress or improving their physical and mental well-being (40/67, 59.7%). However, 49.2% (32/65) strongly agreed or agreed that the eHealth resources were positive. In terms of how eHealth improved quality of life, 64.2% (43/67) were neutral as well as for negativity of the online resources (36/67, 55.4%), not encouraging (33/65, 50.8%), not helpful (34/65, 52.3%), and not relevant (33/67, 50.8%). In contrast, the majority of participants in the Text4Support group strongly agreed or agreed that the messages were on the topic (105/129, 81.4%), were on point (104/129, 80.6%), supportive (105/129, 81.4%), positive (114/129, 88.8%), and helped them cope with loneliness (52/129, 40.3%), stress (57/129, 44.2%), or improve their overall mental well-being (66/129, 51.2%). In terms of how the messages improved quality of life, 42.6% (55/129) in the Text4Support group were neutral and strongly disagreed or disagreed with the messages being negative (112/129, 86.8%), not encouraging (101/129, 78.3%), not helpful (98/129, 76%), and not relevant (86/129, 66.7%). The results revealed statistically significant differences between groups, indicating that the observed effects were not due to chance and were not coincidental.

## Discussion

### Principal Findings

This study provides new insights into how patients perceive text message interventions, eHealth resources, and their engagement with the Text4Support program and the Nova Scotia Health online resources. Findings reveal that the overall mean (SD) satisfaction score in the control group was 5.1 (2.3), and the overall mean (SD) satisfaction score for the Text4Support group was 7.1 (2.2). Compared to the control group, participants in the Text4Support group reported greater engagement and positive program impact. While 53.8% (70/130) of Text4Support recipients always read the messages, only 8.7% (6/69) of the control group always read the eHealth resources. Participants allocated to the Text4Support group were reported to sometimes take action upon reading the messages (42.3% vs 33.3%). A significantly higher proportion of Text4Support users strongly agreed or agreed that the messages were supportive (81.4% vs 41.5%), positive (88.4% vs 49.2%), and helpful in coping with stress (44.2% vs 11.9%), loneliness (40.3% vs 13.4%), and improving mental well-being (51.2% vs 17.9%). In contrast, responses from the control group were largely neutral. The majority of respondents in the Text4Support group agreed or strongly agreed that the messages had a positive impact on them, which achieved statistical significance (*P*<.001) when compared with the e-mental health resource group; however, physical well-being was neutral across the 2 groups and not statistically significant (*P*=.1).

The response rates observed in this study provide important insights into participant engagement with digital mental health interventions. The Text4Support group demonstrated a notably higher response rate (33%) compared to the control group (18%). Although the response rates remain relatively low, raising concerns about potential attrition bias and the representativeness of the findings, the differences suggest that supportive SMS text messaging interventions may play a significant role in enhancing participant engagement and willingness to interact with mental health research and services. Improved response rates have important implications for the validity and generalizability of study findings. Higher response rates enhance the appropriateness and representativeness of the sample [24].

The findings indicate a high level of satisfaction among participants in the Text4Support group, with an average score of 7.1. Although our study participants expressed high satisfaction, other studies recorded higher satisfaction with the supportive text intervention [22,25-27]. For example, a study conducted among educators showed a mean satisfaction of about 8.5 among administrative educators [26]. Again, a cross-sectional study among 2032 participants recorded 8.55 overall mean satisfaction with the Text4Hope program [27]. Among the control group, an overall average satisfaction rate of 5.1 for online mental health resources suggests moderate user satisfaction. In terms of context and use, this measure aligns with the literature in that satisfaction with online mental health services is low to moderate [28]. As an example, one study making use of the Client Satisfaction Questionnaire-8 found that most patients reported low-to-moderate satisfaction with online psychiatric services [28].

The Text4Support program produced significant participant engagement, as 53% of participants consistently read text messages, while only 8.7% of control group members always read online resources. Outcomes from various studies have shown that interactive text-based interventions help maintain long-term therapeutic engagement, which delivers essential immediate mental health support [22,27,29,30]. A systematic review and meta-analysis found that SMS text messaging interventions for health promotion had widespread acceptance among participants and demonstrated high engagement rates throughout different health areas [29,30]. Although few participants always read the messages/eHealth resources and took action afterward, 73.8% and 83.8% of participants in the control and intervention groups, respectively, at least rarely read and took action in our study. However, the relatively low percentage of participants taking consistent action based on the messages suggests that passive consumption may not suffice for significant behavioral change. Integrating interactive components, such as bidirectional messaging, could enhance effectiveness. A scoping review of text message interventions in adolescent mental health services found that 65% of studies involved bidirectional messaging, indicating its potential to boost engagement and outcomes [31].

On the other hand, engagement with the provided eHealth resources was lower, with only a subset of participants actively utilizing them. Research indicates that eHealth interventions often struggle with uptake unless seamlessly integrated into daily routines [32]. Barriers to eHealth engagement usually stem from issues such as lack of time, perceived irrelevance, and digital literacy challenges [33]. Unlike text messages, which require minimal effort to consume, accessing eHealth resources demands additional steps that may deter some users. The brevity of text messages (limited to 160 characters) makes them easier to process, especially for individuals experiencing mental health challenges [34]. In contrast, studies on guided digital interventions suggest that providing step-by-step instructions or integrating behavioral prompts within text messages can enhance the utilization of eHealth resources [35]. One notable finding is that while 66.7% of the Text4Support group and 29.2% in the control group found the messages relevant, 9.3% and 20%, respectively, did not. The Text4Support group exhibited a notably higher percentage of perceived relevance, indicating that Text4Support was more effectively aligned with participants’ mental health needs than the control condition. A significant percentage of participants, 9.3% in the Text4Support group and 20% in the control group, indicated that the messages/online resources were not pertinent to their needs. This disparity highlights the necessity of customizing digital interventions to individual users’ specific needs, circumstances, and preferences. The control group’s elevated percentage of irrelevance likely indicates the generic nature of nontargeted messages, while the Text4Support group’s reduced percentage implies a degree of alignment with participants’ needs, although there is still potential for enhancement.

Regarding perceived relevance and utility, the results show that most Text4Support participants (81.4%) found the messages relevant to the topic. In comparison, 46.2% strongly agreed or agreed with the online resource, underscoring the importance of tailored content. Personalization has been identified as a key factor in the success of text-based interventions. Literature has highlighted that those interventions allowing rapid, personalized exchanges, akin to natural conversation, were more effective in addressing individual needs [22,36].

The purpose of Text4Support was to provide emotional support through daily messages. The Text4Support group overwhelmingly found the messages supportive (81.4%) and positive (88.4%). These findings demonstrate that digital interventions with positive reinforcement significantly improve users’ mental well-being [10,22,37,38]. Research indicates that such interventions can effectively develop therapeutic connections, meeting unmet needs for mental health care [10,27]. However, the debate persists on whether supportive messaging alone yields lasting psychological improvements. Studies suggest combining SMS text messaging with other treatment modalities, such as CBTs, may enhance effectiveness [39]. The supportive text messages delivered in this study were an addition to the patient’s usual care, making it more effective.

The supportive text messages positively impacted mental well-being, with 51.2% of Text4Support participants reporting improvements, compared to 17.9% in the control group. Similarly, 40.3% of the intervention group reported reduced loneliness and 44.2% experienced lower stress levels. Other studies align with our findings; for example, a similar program, Wellness4Teacher, reported that 95.4% of its respondents reported that the messages were at least often positively impactful, while 93.3% reported that the messages were affirming [26]. These findings highlight the potential of digital interventions as supplementary tools for mental health support [40,41].

Our results show substantial differences in neutral responses across all groups in terms of improving the quality of life. The highest proportion of neutral participant responses was 64.2% in the control group and 42.6% in the Text4Support group, totaling 50% among the study sample. Participants who gave neutral responses were all neither for nor against the intervention having an effect on their quality of life. The slightly larger percentage of indifferent responses from the control group indicates either a lack of participation or ineffectiveness of regular care in significantly changing the participants’ assessments of life quality. This is perhaps due to a more focused and structured approach, which is likely to impress participants more than those of general care. The findings align with previous research showing that participants’ opinions about digital treatments vary [42,43]. For example, Proudfoot et al [43] found that while 68% of respondents indicated considerable improvements to their mental health following digital treatments, about 25% reported neutral or unsure answers, underscoring that outcomes are determined contextually. A qualitative research study also documents instances of most respondents feeling neutral or holding dual views about digital mental health interventions [44].

Similarly, a meta-analysis of RCTs highlighted that, as a result of the differences in people’s participation and expectations, neutral answers often emerge in studies involving smartphone-based mental health therapies [42].

Across groups, participants demonstrated similar disagreement percentages, with the coping of stress registering 28.4% in the eHealth resource group and 19.4% in the Text4Support group, resulting in an overall rate of 22.4%. Although digital interventions may have received more positive feedback, they were not effective for everyone, and some participants found them ineffective in stress coping [45].

However, both interventions had a weaker effect on physical well-being, with no significant differences between groups [46,47]. This finding is consistent with prior research, which has shown that text-based interventions can effectively improve mental health outcomes but have a limited influence on physical health behaviors [47,48]. Digital interventions targeting physical health require more structured behavior-change strategies, such as goal setting and reinforcement mechanisms, rather than passive messaging alone [49].

### Implications for Policy, Practice, and Future Research

The findings of this study have several important implications for the design, implementation, and scaling of digital mental health interventions. First, the significantly higher satisfaction and engagement levels observed in the Text4Support group underscore the value of incorporating supportive, brief, and personalized messaging into routine mental health care. Policymakers and health care providers should consider integrating text-based interventions as a standard adjunct to traditional care, particularly for populations that may face barriers to in-person or web-based services.

Given the low engagement with static eHealth resources, health systems should prioritize low-barrier, mobile-first tools that align with users’ daily routines. Moreover, to increase the effectiveness of such interventions, future policy should encourage the development of interactive, bidirectional messaging systems that foster active participation and allow for real-time support and personalization based on user feedback and needs. Tailoring content to individual needs, such as demographic or diagnostic profiles, can further enhance relevance and efficacy, as evidenced by the higher perceived relevance and positive impact of the Text4Support messages.

Although Text4Support currently relies on preprogrammed, therapist-developed messages, future iterations of supportive SMS text messaging interventions could leverage artificial intelligence, including generative artificial intelligence, to enhance message compilation, personalization, and responsiveness. Finally, given the limited impact on physical well-being, digital programs should integrate behavior change techniques (eg, goal setting, reminders, and progress tracking) when addressing physical health outcomes. Health authorities should support cross-sector collaboration between digital health developers, clinicians, and researchers to ensure interventions are both evidence-based and contextually responsive. These findings advocate for a patient-centered, adaptive approach to mental health support, where digital tools are not merely supplemental but are integral to comprehensive, accessible, and scalable care strategies.

### Limitations

Our study has limitations that need to be considered when appraising the results. First, the generalizability of the results may be affected by the comparatively small sample size of participants who completed the follow-up satisfaction survey, particularly in the control group. The imbalance in group sizes (130 in the Text4Support group vs 69 in the control group) may have introduced bias or limited the statistical power to detect differences in some outcomes. The small sample size may be attributed to the online nature of the surveys; literature has reported that online distributed surveys are unlikely to keep participants in a study for follow-up assessment compared to paper-based surveys [50]. One notable limitation of this study is the relatively low survey completion rate. While this may impact the generalizability of the findings, it is consistent with patterns observed in other digital mental health and eHealth trials, which frequently report attrition rates exceeding 30% [51,52]. Again, participants’ satisfaction, engagement, and perception were self-reported, which may result in recall bias since participants may overestimate their feedback. Lastly, the web-based questionnaire used to assess participant satisfaction and perception was not a validated instrument, which may reduce the reliability of the reported satisfaction rates. Nonetheless, this was one of the first RCTs examining user satisfaction among Text4Support users and Nova Scotia mental health and addiction online resources users in Nova Scotia.

### Conclusions

This study’s findings demonstrate the meaningful impact of personalized, supportive SMS text messaging in enhancing engagement, emotional well-being, and satisfaction among mental health service users. As mental health systems evolve to meet rising demands, digital interventions like Text4Support represent a promising, scalable solution that can complement existing care. To maximize their potential, policies must prioritize accessibility, personalization, and integration with broader care models, ensuring that digital tools meet people where they are, with the right message at the right time.

## Supplementary material

10.2196/76587 Checklist 1 CONSORT-eHEALTH (v 1.6.1) checklist.

## Abbreviations

CBT: cognitive behavioral therapy
RCT: randomized controlled trial
REDCap: Research Electronic Data Capture
